# Hydrogen Sensing Performance of ZnO Schottky Diodes in Humid Ambient Conditions with PMMA Membrane Layer

**DOI:** 10.3390/s20030835

**Published:** 2020-02-04

**Authors:** Soohwan Jang, Sunwoo Jung, Kwang Hyeon Baik

**Affiliations:** 1 Department of Chemical Engineering, Dankook University, Yongin 16890, Korea; jangmountain@dankook.ac.kr (S.J.); cmkoung12@dankook.ac.kr (S.J.); 2 Department of Materials Science and Engineering, Hongik University, Sejong 30016, Korea

**Keywords:** gas sensor, ZnO, humidity, hydrogen, Schottky diode

## Abstract

Enhanced hydrogen sensing performance of Pt Schottky diodes on ZnO single crystal wafers in humid ambient conditions is reported using a polymethylmethacrylate (PMMA) membrane layer. ZnO diode sensors showed little change in forward current when switching to wet ambient H_2_ conditions with 100% relative humidity. This sensitivity drop in the presence of water vapor can be attributed to surface coverage of hydroxyl groups on the Pt surface in humid ambient conditions. The hydrogen sensitivity of PMMA-coated diode sensors recovered up to 805% in wet H_2_ ambient conditions at room temperature. The PMMA layer can selectively filter water vapor and allow H_2_ molecules to pass through the membrane layer. It is clear that the PMMA layer can effectively serve as a moisture barrier because of low water vapor permeability and its hydrophobicity. In both dry and wet conditions, ZnO diodes exhibited relatively fast and stable on/off switching in each cycle with good repeatability.

## 1. Introduction

Gas sensors based on metal oxide (MOX) semiconductors have been widely used for toxic gas detection and quality air control. Extensive efforts have been devoted to developing highly sensitive, selective, reliable, stable, and low-cost sensors [1,2,3,4,5,6,7]. Various MOXs such as SnO_2_, TiO_2_, ZnO, WO_3_, NiO, and Ga_2_O_3_ have shown significant change in the conductance through surface interaction and charge transfer between gas molecules and oxide surface atoms. Among those MOXs, ZnO is highly promising for the potential use for gas sensors due to high conductance change, thermal and chemical stability, and facile synthesis into nanostructures having high surface-to-volume ratios [8,9,10,11,12]. Surface modification with ZnO nanorods and doping with specific dopants have yielded dramatic increases of its sensitivity and selectivity in gas sensor applications [13,14,15]. 

One of the most challenging issues for highly precise gas sensors is the selectivity, which detects the specific gas molecules under other reducing gas mixtures as well as in humid ambient conditions. In the presence of water vapor, SnO_2_-based gas sensors have shown the increased sensitivity as well as degraded sensor signals over a certain period of time [16,17,18]. The increase in operation temperature and the addition of select dopants were proposed to mitigate the effects of water on the sensitivity [19,20]. In the case of diode-based gas sensors, the sensitivity and selectivity were reported to be severely degraded in humid air conditions [21,22,23]. Under the exposure of water vapor, the surface-active sites for gas adsorption can be blocked by water molecules, thus resulting in the sensitivity drop in the presence of water vapor. There are rapidly growing demands for stable gas sensors to detect a small amount of hydrogen in extreme conditions, especially in hydrogen-fueled vehicles that require conditions above 50% relative humidity (RH) at low temperatures to operate. Several methods have been proposed to solve the humidity problem in gas sensing, including polymer-based membrane layers such as polyvinyl fluoride, polytetrafluoroethylene, polymethylmethacrylate (PMMA), and polyimide, as well as inorganic membranes [24,25,26,27]. The permeability and its selectivity of polymer membrane are significantly influenced by various factors, such as the polarity of membrane materials, the degree of crystallization, and glass transition temperatures. Non-polar polymers exhibit a lower permeability to water vapor and polar gas molecules. Thus, a hydrophobic PMMA layer can serve as a selective filter due to its low permeability coefficient for moisture to water vapor by reducing water adsorption on the oxide surface. In this paper, we investigated the hydrogen sensing performance in high humidity conditions using PMMA protective layers directly coated on Pt/ZnO Schottky diode sensors. 

## 2. Materials and Methods

**Figure 1:**
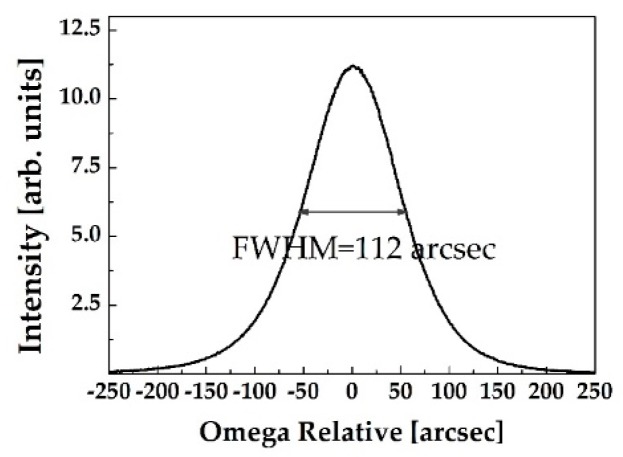
The relative *w*-rocking curve of *c*-plane (0001) ZnO single crystal wafer showing the full-width at half maximum (FWHM) value of 112 arcsec.

The 5 mm × 5 mm ZnO single crystal substrates with the *c*-axis (0001) direction were purchased from a commercial supplier. Both substrates were double sided polished to a thickness of 0.5 mm. Undoped ZnO substrates were characterized by scanning electron microscopy, atomic force microscope, and high-resolution X-ray diffraction analysis. The root-mean-square roughness of the ZnO substrates was measured to be in the range of 1–5 nm with a scan size of 5 × 5 μm^2^. As can be seen in Figure 1, the full width at half maximum of the X-ray rocking curve of the (0001) ZnO single crystal was measured to be 112 arcsec, indicating high crystalline quality. The electron carrier concentration of ZnO single crystal wafers was measured to be ~5 × 10^17^ cm^−3^. Ti/Al (20/100 nm) metal layers were evaporated on ZnO substrates as Ohmic contact, formed by a photolithography and lift-off process. The Ohmic metallization was achieved by annealing at 300 °C for 1 min in ambient nitrogen in a rapid thermal annealer. Then, 200 nm-thick Si_3_N_4_ was deposited by plasma-enhanced chemical vapor deposition for the passivation layer. Wet etching with buffered HF solution was used for window opening for the active gas sensing surface. Schottky contact was formed by the evaporation of the catalytic Pt layer with 10 nm thickness. Ti/Au (20/100 nm) were evaporated on both Schottky and Ohmic contacts for metal probe pads. The current–voltage (I–V) characteristics were recorded using an Agilent 4155C semiconductor parameter analyzer (Agilent technologies, Santa Clara, CA, USA) under various H_2_ concentrations balanced with nitrogen. In order to prepare the PMMA protective layer on the device, PMMA with a molecular weight of 996,000 was dissolved in solvent (anisole) with a concentration of 40 mg/mL, followed by mixing of the solution for 12 h. PMMA was spin coated at 4000 rpm for 30 s, resulting in a thickness of 145 nm. The morphologies of the PMMA surface and cross-section were observed using a scanning electron microscope, as shown in the author’s previous literature [22]. The contact angle measurement for the PMMA dissolved in anisole on the Pt surface was 15.91°, whereas that on Si was 68.34°, clearly indicating that PMMA was more hydrophilic on the Pt layer. The hydrogen gas responses of Schottky diodes with and without PMMA membrane layers were measured when loaded in a gas test chamber with 4% dry H_2_ in ambient nitrogen. A water bubbler was utilized to produce wet ambient H_2_ for humidity conditions with 100% RH.

## 3. Results and Discussion

**Figure 2:**
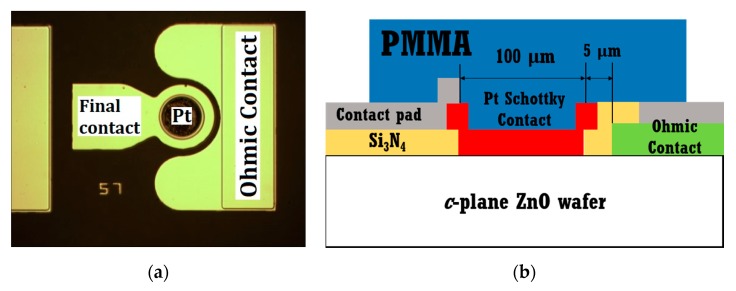
(**a**) Top-view optical microscope image of fabricated Schottky diode sensor; (**b**) cross-sectional schematic of polymethylmethacrylate (PMMA)-coated Pt/ZnO Schottky diode.

Figure 2a shows the top-view optical microscope image of the fabricated Schottky barrier diode (SBD) on a ZnO single crystal wafer. Figure 2b presents the schematic cross-section of the PMMA-coated diode on the catalytic Pt surface with Ohmic contact and a Si_3_N_4_ passivation layer. 

**Figure 3:**
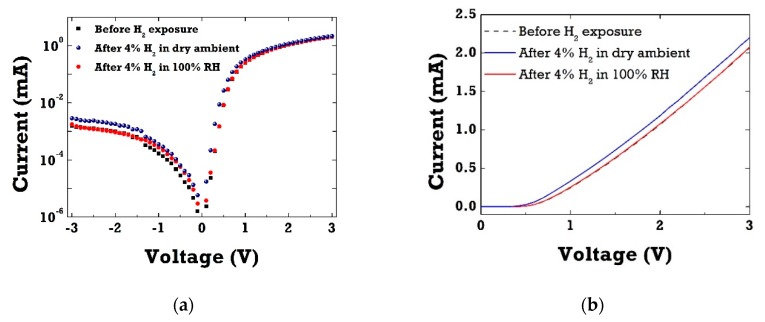
(**a**) Current–voltage (I–V) curve of ZnO diodes in log-scale before and after 4% hydrogen exposure in dry and wet ambient conditions; (**b**) corresponding I–V characteristics of ZnO diodes in linear-scale before and after 4% hydrogen exposure in dry and wet ambient conditions.

Figure 3a shows current–voltage (I–V) characteristics in-log scale of Pt/ZnO Schottky barrier diodes (SBD) before and after 4% H_2_ exposure in dry ambient as well as in wet ambient conditions. As clearly seen in Figure 3b of I–V curves in linear scale, Pt/ZnO SBDs exhibited excellent response to hydrogen at room temperature. Under H_2_ exposure, hydrogen gas molecules adsorbed on the Pt catalytic layer, thus leading to the increase in forward current by reducing the effective Schottky barrier height (SBH). When switching to wet ambient conditions (100% RH), little change in forward current was observed upon H_2_ gas exposure. This sensitivity drop in the presence of water vapor can be attributed to surface coverage of hydroxyl groups on the Pt surface in humid ambient conditions. As water vapor chemisorbed on the Pt catalytic surface, it resulted in the decrease of surface adsorption sites available for hydrogen. The cross-sensitivity toward water vapor has been well reported in SnO_2_-based MOX gas sensors [18,19,20]. Water vapor has been shown not only to decrease the resistance of SnO_2_-based materials, but also to significantly interfere with most target gases, resulting in the selectivity issues of gas sensors in humid air.

**Figure 4:**
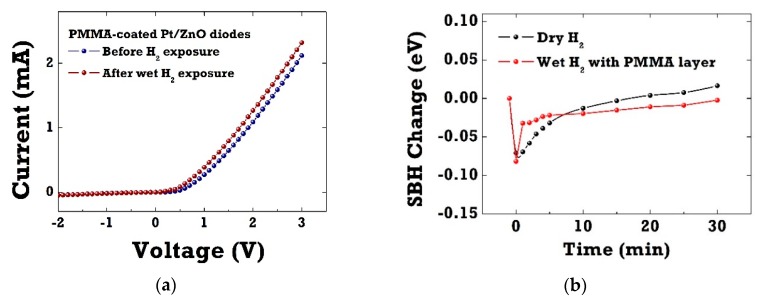
(**a**) I–V characteristics of PMMA-coated Pt/ZnO SBDs before and after wet 4% hydrogen exposure; (**b**) the transient change in Schottky barrier height (SBH) of Pt/ZnO SBDs in dry H_2_ and PMMA-coated Pt/ZnO diodes in wet H_2_ ambient conditions.

Figure 4a shows the I–V characteristics of Pt/ZnO SBD diodes with the PMMA membrane layer before and after 4% H_2_ exposure in wet ambient conditions (RH 100%). The hydrogen sensitivity of PMMA-coated diode sensors in wet H_2_ almost recovered to the same level with the one in dry H_2_ ambient conditions. The relative current change for PMMA-coated diode sensors reached the maximum value of 805% in wet H_2_ ambient conditions at room temperature. The PMMA layer performed well in ZnO SBDs as a membrane layer, which selectively blocked water molecules, as previously reported by the authors [21,22]. Figure 4b presents the transient change in SBH of Pt/ZnO SBDs in dry H_2_ and PMMA-coated Pt/ZnO diodes in wet H_2_ ambient conditions. Note that the SBH values were extracted from the I–V curves using the thermionic emission model. The SBH changes were measured to be 72–75 meV for both SBD diodes upon exposure to hydrogen. After switching back to ambient nitrogen, the SBH of PMMA-coated diodes showed faster recovery. 

**Figure 5:**
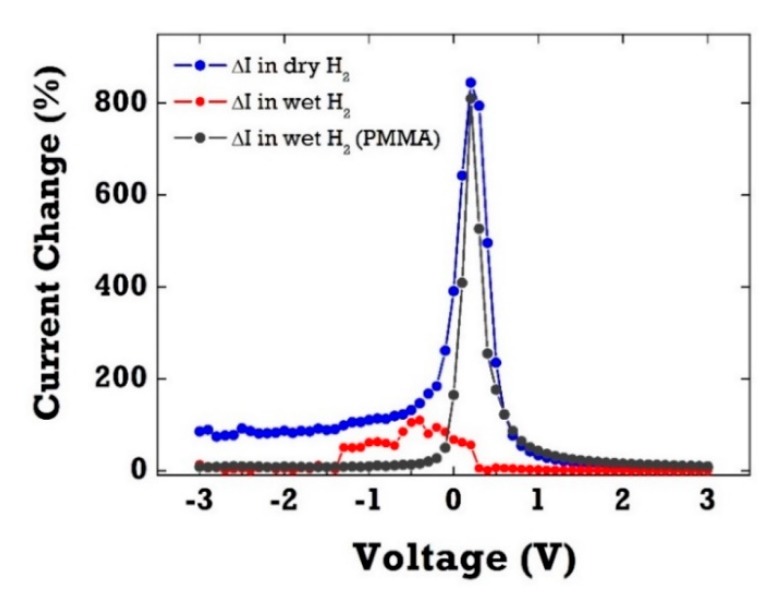
The current change as a function of bias voltage of ZnO diode sensors with and without the PMMA membrane layer in dry and wet H_2_ exposure. H_2_ (4%) was injected into the gas chamber for 30 s.

Figure 5 shows the relative current change (ΔI) in percentage of PMMA-coated Pt/ZnO SBDs with and without the PMMA membrane layer upon exposure to 4% H_2_ in dry and wet ambient conditions (100% RH). Clearly, the SBD diodes without any protective layer exhibited only a negligible current response to 4% H_2_ in wet conditions (RH 100%). This H_2_ sensitivity drop in the presence of water vapor can be attributed to surface coverage of hydroxyl groups on the Pt surface in humid ambient conditions. As water vapor chemisorbed on the Pt catalytic surface, it resulted in the decrease of surface adsorption sites available for hydrogen. It suggests that there is competition and strong interference between water vapor and H_2_ for the active adsorption sites. The sensitivity, the relative current change as a percentage, is defined as ΔI [$(I_{H_{2}}$–$I_{N_{2}}$)/${\;I}_{N_{2}}$] in percentage where $I_{H_{2}}$ and $I_{N_{2}}$ denote diode currents measured in H_2_ and N_2_ ambient, respectively. The water-induced effect has been well established in metal oxide-based gas sensors. Water vapor has been shown not only to decrease the resistance of SnO_2_-based materials, but also to significantly interfere with most target gases, resulting in selectivity issues of gas sensors in humid air. In contrast, PMMA-coated SBD diodes demonstrated little change in sensitivity up to 800% at 0.2 V in high humid condition. The PMMA layer can selectively filter water vapor and allow H_2_ molecules to pass through the membrane layer. H_2_ molecules with small kinetic diameter (2.89 Å) can permeate easily thorough the PMMA layer when compared with other gases, such as CO_2_ (3.3 Å), N_2_ (3.64 Å), and CH_4_ (3.8 Å). 

**Figure 6:**
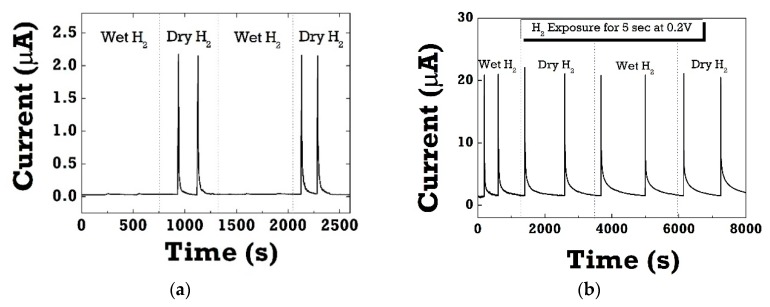
(**a**) Cyclic response curves of Pt/ZnO diode sensors without PMMA layer; (**b**) cyclic response curves of PMMA-coated Pt/ZnO diode sensors, which were measured at 0.2 V forward voltage when switching H_2_ on and off in dry and wet ambient conditions. The same level of sensitivity was observed in PMMA-coated diode sensors even in repeated cycles of switching dry and wet H_2_ ambient conditions in 100% RH.

Figure 6a shows the cyclic response of the Pt/ZnO Schottky diode upon switching 4% H_2_ on and off in dry and wet ambient conditions. One can see a dramatic decrease in forward currents in wet H_2_ ambient conditions measured at 0.2 V at room temperature. The inset figures show the negligible current response to hydrogen in the range of a few nA. It suggests that there would be competition and strong interference between water vapor and H_2_ for the adsorption sites. Water molecules significantly interfere with most target gases, resulting in the selectivity issues of gas sensors in humid air. In the case of PMMA-coated diodes, the hydrogen sensitivity in wet H_2_ recovered to the same level in dry H_2_ ambient conditions as can be seen in Figure 6b. Clearly, the PMMA layer could effectively act as effective moisture barrier because of low water vapor permeability. In both dry and wet conditions, SBD diodes exhibited relatively fast and stable on/off switching in each cycle with good repeatability. 

## 4. Conclusions

Pt/ZnO Schottky diode sensors showed a drastic decrease of hydrogen sensitivity in the presence of water vapor. This sensitivity drop in the presence of water vapor resulted from the surface coverage of hydroxyl groups on the Pt surface, thus leading to the interference of surface adsorption sites available for hydrogen. By employing a PMMA membrane layer on the catalytic Pt surface, enhanced hydrogen sensing performance was achieved in humid ambient at room temperature. The hydrogen sensitivity up to 805% was measured in 100% relative humidity. The PMMA-coated diode sensors also showed stable sensor operation in repeated cycles of H_2_ on and off without any degradation. PMMA membrane layers can be effectively integrated on Pt surface of diode sensor and work as a selective filter of water molecules in humid ambient conditions.
